# A retrospective analysis of breast cancer subtype based on ER/PR and HER2 status in Ghanaian patients at the Korle Bu Teaching Hospital, Ghana

**DOI:** 10.1186/s12907-015-0014-4

**Published:** 2015-07-09

**Authors:** Bernard Seshie, Nii Armah Adu-Aryee, Florence Dedey, Benedict Calys-Tagoe, Joe-Nat Clegg-Lamptey

**Affiliations:** Department of Surgery, Tema General Hospital, Tema, Ghana; Department of Surgery, School of Medicine and Dentistry, University of Ghana, Accra, Ghana; Department of Community Health, School of Public Health, University of Ghana, Accra, Ghana

**Keywords:** Breast cancer, Subtype, ER, PR, HER2

## Abstract

**Background:**

Breast cancer is a heterogeneous disease composed of multiple subgroups with different molecular alterations, cellular composition, clinical behaviour, and response to treatment. This study evaluates the occurrence of the various subtypes and their clinical and pathological behaviour in the Ghanaian breast cancer population at the Korle Bu Teaching Hospital (KBTH).

**Methods:**

Retrospective review of case notes of patients who had completed treatment for breast cancer at the KBTH within the last 5 years was conducted between April 2011 and March 2012. Subtypes were determined by immunohistochemistry classification based on expression of estrogen receptor (ER), progesterone receptor (PR), and human epidermal growth factor receptor-2 (HER-2).

**Result:**

A total of 165 cases contributed to this study. The mean age at diagnosis was 52.5 ± 12.1 years. Tumour size ranged from 0.8 cm to 15 cm with a mean of 4.9 ± 2.8 cm and median of 4 cm. Tumour grade was Grade I 8.3 %, Grade II 60.8 % and Grade III 30.8 %. ER, PR and HER2/neu receptor positivity was 32.1, 25.6 and 25.5 % respectively. Almost half (49.4 %) of the study population had triple negative tumours. Luminal A, luminal B and non-luminal HER2 were 25.6, 12.2, and 12.8 % respectively. No statistically significant association was seen between subtype and tumour size, tumour grade, lymph node status and age at diagnosis.

**Conclusion:**

Triple negative tumour is the most occurring subtype in the Ghanaian breast cancer population treated at the Korle Bu Teaching Hospital. Lack of association seen between subtypes and their clinical and pathological behaviour could be due to small sample size.

## Background

Breast cancer is still the most common cancer in women comprising 16 % of all female cancers worldwide [1]. With increasing improvement in treatment modalities like hormonal and chemotherapy, however, mortality has declined [2]. But this decline is faster in white Americans compared to black Americans in the United States of America, although the incidence of breast cancer is lower in the latter [3]. The poorer prognosis in blacks has been attributed to a number of factors, including the observation that blacks appear to be at higher risk of breast cancer at an early age, and are diagnosed with more aggressive and advanced tumours [4, 5]. In Ghana, where more than 50 % of patients present with locally advanced or metastatic disease, 5-year survival was reported as only 25.3 % in 2001 [6].

It is now clear that breast cancer is a heterogeneous disease of multiple subgroups with different molecular alterations, cellular composition, clinical behaviour, and response to treatment [7–9]. Hence, standard clinical prognostic features such as age, tumour size, nodal status, grade, and hormone receptor status may be inaccurate. Consequently, many patients are perhaps given treatment they may not need and benefit from. On the other hand, the true risk in some patients is underestimated and some may be given false assurances of favourable prognosis [10].

Several studies have attested to the higher prevalence of triple negative tumours with poorer prognosis in breast cancer patients of African origin [5, 11], although a study from Nigeria reported no difference in the pattern of hormone receptors in the African breast cancer population compared to other populations [12].

This study was undertaken to determine the occurrence of the various subtypes of breast cancer in Ghanaian patients seeking treatment at the Korle Bu Teaching Hospital and to determine the clinical and pathological behaviour of the different subtypes (grade, tumour size, lymph node burden and age at diagnosis)

## Methods

Data for this study was from an ongoing study on upper limb morbidity following treatment of breast cancer in Ghana, which has been approved by the Ethical and Protocol Review Committee, University of Ghana School of Medicine and Dentistry.

### Study population

Korle Bu Teaching Hospital (KBTH) is the largest teaching hospital in Ghana, the leading tertiary hospital and the major referral centre in the country. It also serves as the teaching hospital of the University of Ghana School of Medicine and Dentistry

Breast Cancer patients who had received and completed treatment for breast cancer at the Korle Bu Teaching Hospital (KBTH) within the last 5 years and were being seen for out-patient review constituted the study population. Data was thus collected between April 2011 and March 2012. During the period 363 consecutive patients who met the above criteria were seen and their case notes reviewed. Immunohistochemistry (IHC) for estrogen receptor (ER), progesterone receptor (PR), and HER-2/neu, which is a prerequisite for this study, was available for 165. They thus constituted the subset for this study. Demographic information (hand dominance and educational level), breast cancer clinico-pathological features (age at diagnosis, tumour size, tumour grade, lymph node status, hormonal receptors status) and treatment modality (type of surgery, chemotherapy) were extracted from the case notes.

Pathology reports from which ER, PR and HER-2/neu, were obtained came from Korle-Bu Teaching Hospital. IHC was performed on formalin-fixed paraffin embedded tissue sections. The ER and PR tests were scored based on an aggregate score of percentage of tumour stained and staining intensity. Aggregate score of more than 2 were considered positive; that is, a minimum of 1–10 % stained associated with minimum intensity. HER-2/neu was considered positive if an IHC 3+ result was found. Flourescence in situ hybridization (FISH) was not available in the institution.

For this study we used Immunohistochemistry (IHC) classification that categorizes tumours according to the expression of estrogen receptor (ER), progesterone receptor (PR), and HER-2/neu. Expression of basal cytokeratin 5/6 and EGFR were not determined in these cases. Hence the triple negative tumours included both core basal phenotype, equivalent to the basal-like by gene expression profiling, and five negative phenotype

Below is the categorization used:

- Luminal A (ER/PR+, HER2-)
  - ER+/PR+/HER2-; ER-/PR+/HER2-; or ER+/PR-/HER2-
- Luminal B (ER/PR+, HER2+)
  - ER+/PR+/HER2+; ER-/PR+/HER2+; or ER+/PR-/HER2+
- Non-luminal HER2 (ER-/PR-/HER2+)
  - ER-/PR-/HER2+
- Triple Negative (ER-/PR-/HER2-)
  - ER-/PR-/HER2-

Histological grading was by the Bloom-Richardson grading system that combined scores for nuclear grade, tubule formation and mitotic rate [13].

### Statistical analysis

SPSS 16.0 was used for the descriptive data analysis. To test for association between subtype and tumour grade, and subtype and lymph node burden contingency table was used and Chi Square test done. One-way ANOVA was conducted to compare the differences in tumour size and age at diagnosis between breast cancer subtypes.

## Results

**Fig. 1:**
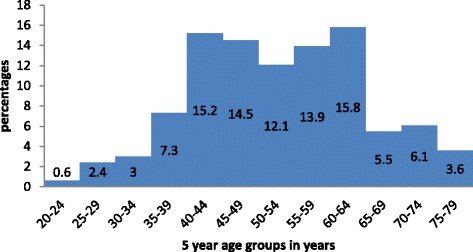
Age distribution of study patient

**Fig. 2:**
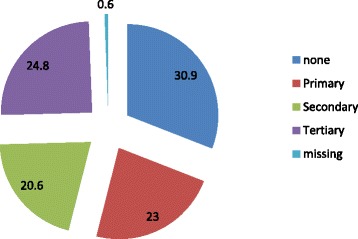
Educational level of study patients

A total of 165 cases contributed to this study. The mean age at diagnosis was 52.5 ± 12.1 years. The youngest patient in the study group was 24 years and the oldest person was 77 years at the time of diagnosis. Figure 1 shows the age distribution at the time of diagnosis. The educational level of the study population is as shown in Fig. 2.

In 50.9 % of the study population the tumour was located in the left breast with the remaining 49.1 % in the right breast. Over 90 % of the patients were right handed. There was however, no correlation between hand dominance and tumour site (Spearman’s correlation value of 0.034, p-value of 0.666).

Tumour size ranged from 0.8 cm to 15 cm with a mean of 4.9 ± 2.8 cm and median of 4. Eight tumours were ≥10 cm. Tumour size (T in TNM classification) values were available for 155 cases. T1 (maximum diameter 2 cm or less) tumour was present in 17/155 (11 %) and T2 (size more than 2 cm but not more than 5 cm), T3 (bigger than 5 cm) and T4 (spread to chest wall, or skin—including inflammatory cancer) tumours were 71 (45.8 %), 42 (27.1 %), and 25 (15.2 %) respectively.

**Fig. 3:**
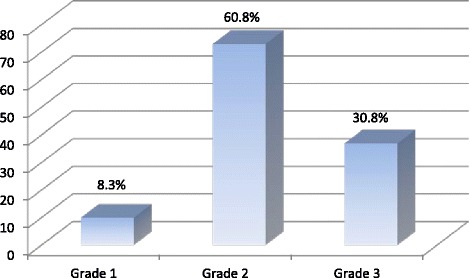
Histological grade of tumours

The majority of tumours were Grade 2. The distribution of tumour grade is shown in Fig. 3.

Mastectomy was done in 97.6 % (161/165) of cases. Only 4 cases (2.4 %) had breast conservation surgery (BCS). Apart from 2 patients who had sentinel node biopsy, all the other patients had axillary clearance. Less than 10 lymph nodes were removed in 52.2 % of cases. Mean lymph node (LN) removal was 9.4 ± 4.3 and mean LN involvement was 3.3 ± 3.6. Regional LN involvement (TNM) in 137 patients in which data was available was N0 47 (34.3 %), N1 35 (25.5 %), N2 46 (33.6 %) and N3 9 (6.6 %).

ER, PR and HER 2 neu receptor positivity was 32.1 % (53/165), 25.6 % (42/164) and 25.5 % (40/157) respectively. The distribution of the receptor status by tumour size, tumour grade and LN positivity is shown in Table 1.

**Table 1:** Distribution of receptor status by tumour size, grade and LN involvement

Tumour size	Receptor status								
	ER+	ER-	Total	PR+	PR-	Total	Her2+	Her2-	Total
T1	9 (17.6)	8 (7.7)	17 (11.0)	7 (17.5)	9 (7.9)	16 (10.4)	4 (10.8)	12 (10.8)	16 (10.0)
T2	24 (47.1)	47 (45.2)	71 (45.8)	21 (52.3)	50 (43.9)	71 (46.1)	17 (46.9)	49 (44.1)	66 (44.6)
T3	12 (20.6)	30 (28.8)	42 (27.1)	6 (15.0)	36 (31.6)	42 (27.3)	9 (24.3)	33 (29.7)	42 (28.4)
T4	6 (11.8)	19 (18.3)	25 (6.1)	6 (15.0)	19 (16.7)	25 (16.2)	7 (18.9)	17 (15.3)	24 (16.2)
Total	51	104	155	40	114	154	37	111	148
Tumour grade									
1	5 (50)	5 (50)	10	4 (40)	6 (60)	10	3 (33.3)	6 (66.7)	9
2	24 (32.9)	49 (67.1)	73	23 (31.9)	49 (68.1)	72	21 (30.4)	48 (69.6)	69
3	8 (21.6)	29 (78.4)	37	5 (13.5)	32 (86.5)	37	8 (22.2)	28 (77.8)	36
Total	37	83	120	32	87	119	32	82	114
Lymph node positivity									
N0	12 (25.5)	35 (74.5)	47	8 (17.0)	39 (83.0)	47	11 (25.0)	33 (75.0)	44
N1	13 (32.1)	22 (62.9)	35	12 (34.3)	23 (65.7)	35	8 (25.0)	24 (75.0)	32
N2	16 (34.8)	30 (65.2)	46	10 (22.2)	35 (77.8)	45	12 (26.1)	34 (73.9)	46
N3	2 (22.2)	7 (77.8)	9	0 (0)	9 (100)	9	3 (33.3)	6 (66.7)	9
Total	43	94	137	30	106	136	34	97	131

**Fig. 4:**
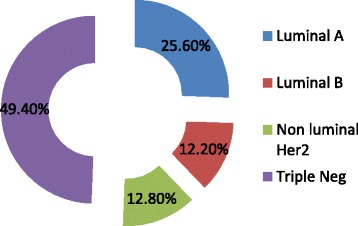
Distribution of various subtypes

Data for breast cancer subtype was available for 156 cases. Almost half (49.4 %) of the study population had triple negative tumours. The distribution of various subtypes is as shown in Fig. 4.

The distribution of the subtype by tumour size, tumour grade and LN positivity is shown in Table 2. Luminal A subtype constituted 53.3 % of T1 tumours, whereas triple negative subtype represented 50 % of T2 tumours, 57.1 % of T3 tumours and 50 % of T4 tumours. However, the difference in tumour size among the subtypes was not significant (F_3, 113_ = 1.26, *p* = 0.262). Regarding tumour grade, 45.5 % of Grade 2 tumours and 52.8 % of Grade 3 tumours were triple negative subtype. But there was no statistically significant association between tumour grade and subtype (p-value = 0.515). Although 51.1 % of N2 and 66.7 % of N3 lymph node status were triple negative subtype, no significant association was seen statistically (p-value = 0.547). The same applied to age at diagnosis (F_3, 152_ = .507, *p =* 0.678)

**Table 2:** Distribution of subtype by tumour size, grade and LN involvement

Subtype					
Tumour size	Luminal A	Luminal B	Non luminal Her2	Triple Neg	Total
T1	8 (53.3)	2 (13.3)	2 (13.3)	3 (20.0)	15 (100)
T2	17 (25.8)	10 (15.2)	6 (9.1)	33 (50.0)	66 (100)
T3	9 (21.4)	4 (9.5)	5 (11.9)	24 (57.1)	42 (100)
T4	5 (20.8)	2 (8.3)	5 (20.8)	12 (50.0)	24 (100)
Total	39	18	18	72	147
Tumour grade					
1	3 (33.3)	2 (22.2)	1 (11.1)	3 (33.3)	9
2	17 (25.0)	10 (14.7)	10 (14.7)	31 (45.5)	68
3	9 (25.0)	1 (2.8)	7 (19.4)	19 (52.8)	36
Total	29	13	18	53	113
Lymph node positivity					
N0	10 (22.7)	4 (9.1)	7 (15.9)	23 (52.3)	44
N1	12 (37.5)	4 (12.5)	4 (12.5)	12 (37.5)	32
N2	10 (22.2)	7 (15.6)	5 (11.1)	23 (51.1)	45
N3	0(0)	2 (22.2)	1 (11.1)	6 (66.7)	9
Total	32	17	17	64	130

In the study population, 43.1 % received between 2 to 6 cycles of neoadjuvant chemotherapy. The commonest combination therapy used as neoadjuvant and adjuvant therapy was Cyclophosphamide—Doxorubicin—5 Fluorouracil (CAF) in 85.5 % of cases. 5-Fluorouracil—Epirubicin—Cyclophosphamide (FEC) 6.2 %, Cyclophosphamide—Methotrexate -5-Fluorouracil (CMF) 6.2 %, and Paclitaxel in only 1.4 %.

## Discussion

In this study of patients treated for breast cancer we found predominance of hormone receptor negative tumours (49.4 %). This is consistent with a study from Kumasi-Ghana between July 2004 and June 2009, which reported 42.5 % triple negative tumours in 54 breast cancer patients [14]. An earlier from the same centre that compared Ghanaian breast cancer patients with black American and white American reported a higher percentage of hormone negative tumours of 82.2 % in Ghanaian women compared to 26.4 and 16.0 % in black American and white American women respectively [11].

DNA microarray analysis of gene expression has identified five subtypes with different gene expression characteristics and differences in behaviour [15]. The usefulness of gene expression pattern rests in its value as a prognostic maker [16]. Although considered as gold standard it has not been widely used due to expense and difficulty using paraffin-embedded material. Hence, the use of immunohistochemisty (IHC) which is simple, workable, and capable of classifying tumours into subtypes which are surrogates to gene expression pattern [17].

**Fig. 5:**
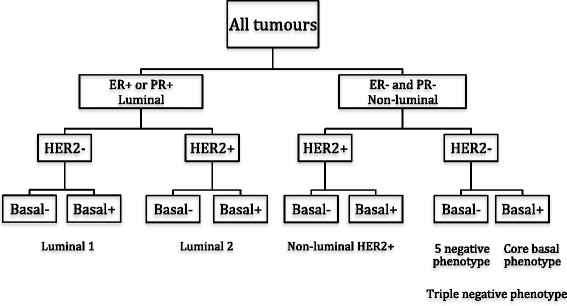
Classification of breast cancer subtype according to IHC marker profile [7]

IHC for hormone receptor status, human epidermal growth factor receptor-2 (HER2) status, and at least one basal marker (cytokeratin [CK]5/6 or epidermal growth factor receptor [EGFR]) enable the division of tumours into Luminal 1, Luminal 2, Non-luminal HER2 positive tumours, and triple negative tumours (Fig. 5) and are associated with different behaviour [7].

Using IHC classification based on expression of ER, PR, and HER-2 receptors tumours were grouped in Luminal A, Luminal B, Non-luminal HER-2 and triple negative tumours. We observed a high prevalence of triple negative subtype. Indeed, almost half of the cases were triple negative. This is consistent with some studies from Nigeria and Senegal in which majority of tumours were basal-like 27 % or unclassified 28 % subtype (surrogate for triple negative) [18]. However, this is in contrast to another study from Nigeria in which majority of tumours were Luminal A (77.6 %) [12]. Luminal A, although the second most frequently occurring subtype in our study, was present in only 25.60 % of cases. One limitation to our study was the non availability of retrospective data on basal markers (cytokeratin [CK]5/6 or epidermal growth factor receptor [EGFR]) as they are not routinely done in our centre. This would have enabled us stratify the triple negative tumours into 5 negative phenotype (non-basal triple negative) and core basal phenotype which many studies had shown to have different behaviour with the latter being more aggressive [5, 8, 17, 19, 20].

In addition, BRCA 1 and 2 mutations have not been determined in our study population. Hence, we are unable to evaluate their contribution to the high prevalence of triple negative tumours in this study. However, several studies have documented high proportion of triple negative breast cancer in carriers of these germ-line mutations, especial BRCA 1 [21–25]. Future research is needed in this area because of its implications for treatment. Triple negative breast cancer patients with BRCA 1 or BRCA like tumours can benefit from treatment with PARP inhibitors [26].

In this study triple negative tumours appeared to be associated with high mean tumour size and higher proportion of T2, T3 and T4 tumours compared to other subtypes. Indeed, 53.3 % of T1 tumours were luminal A subtype whereas over 50 % T2 to T4 tumours were Triple negative tumours. They also constitute greater proportion of Grade 2 and Grade 3 tumours and N2 and N3 tumours. These are consistent with other studies that have shown that triple negative breast cancer has very unfavourable and aggressive clinicopathological features [5, 11]. However, we failed to find significant statistical association.

Our findings have implication for treatment of breast cancer in Ghana. In the past, patient with breast cancer were treated blindly with tamoxifen. However, approximately 50 % of our patients may not be suitable for hormonal or targeted therapy because they are either negative for ER/PR or do not over express HER2. Hence, they will not benefit from the advantages of these modalities of treatment [27–30]. Currently chemotherapy remains the only systemic treatment for this category of patients. Fortunately, core basal phenotype which is a subset of triple negative breast cancer has the greatest short-term effect from cytotoxics compared to all other subtypes. But this cannot be said about the 5 negative phenotype [31]. Several studies using post treatment American Joint Committee on Cancer tumour-node-metastasis staging for invasive carcinoma have documented higher complete pathological response in the core basal phenotype of triple negative breast cancer compared to all other subtypes [5, 31]. However, they still have poorer prognosis due to higher likelihood of relapse in those with residual disease [9, 31].

Several of these studies demonstrated the importance of neo-adjuvant chemotherapy in triple negative breast cancer patients. This is not for the sole purpose of tumour reduction to facilitate surgery, but also to assess response to cytotoxic drugs and predict the likelihood of relapse in patients with residual disease. But in our study only 43.1 % received between 2 to 6 cycles of neo-adjuvant chemotherapy. In our study 85.5 % of patient received CAF either as neo-adjuvant, adjuvant or both despite almost half of the patients being triple negative. However, studies have shown that basal-like and HER2+ subtypes are more sensitive to neo-adjuvant chemotherapy with paclitaxel- and doxorubicin-containing regimes compared to the luminal subtype [9]. Also for the triple negative tumours (especially BRCA—mutated disease), platinum based chemotherapy and PARP inhibitors may hold some promise [32, 33]. The neoadjuvant/adjuvant treatment of the patients in our study therefore appears to be suboptimal.

For surgical treatment, 97.6 % of the patients had mastectomy, a rather high rate compared to what is reported in Europe, North America and Japan (between 27.5 to 64 % [34–37]. Breast conserving surgery was only done in 2.4 % of our patients, a rather low rate as compared to rates of 54 % to over 70 % elsewhere [35–39]. As many as 66 % of the patients in this study presented with a T1 or 2 tumours while almost 60 % had N0 or 1 lymph node staging. Breast conservation surgery may have been suitable for many of these patients. However, almost half of the patients had triple negative subtype and about 91 % had grade 2 and 3 tumours. These factors, as well as increasing tumour size, have been found to be independent predictors of mastectomy but are not contraindications to BCS [34, 36, 38, 40],. Although there is no optimal mastectomy rate [41], evidence suggests that our patients may be presenting late and refusing treatment for breast cancer partly because of the fear of mastectomy. Indeed, in a previous study at the KBTH, fear of mastectomy was the reason for delayed presentation and absconding before and during treatment in 24.2 and 57.2 % of patients respectively [42]. Hence, more breast conservation should be encouraged where indicated.

The limitation of this study was the small sample size. We were thus unable to demonstrate statistically any association between the subtypes and clinical and pathological behaviour. We did not also have information about the menopausal status of the participants to determine the proportions of the various subtypes that were premenopausal.

## Conclusion

Triple negative tumour is the most commonly occurring subtype in the Ghanaian breast cancer population treated at the Korle Bu Teaching Hospital. Hence, blind hormonal therapy is not justifiable. Lack of significant association between subtypes and their clinical and pathological behaviour could be due to the small sample size. We recommend the inclusion of basal makers in the IHC panel on routine basis.

## Abbreviations

KBTH: Korle Bu Teaching Hospital
ER: Estrogen receptor
PR: Progesterone receptor
HER2: Human epidermal receptor 2
IHC: Immunohistochemistry
EGFR: Epidermal growth factor receptor
ESBC: Early stage breast cancer
BCS: Breast conservation surgery
CK: Cytokeratin
CAF: Cyclophosphamide–Doxorubicin–5 Fluorouracil
FEC: 5Fluorouracil–Epirubicin-Cyclophosphamide
CMF: Cyclophosphamide- Methotrexate-5 Fluorouracil
LN: Lymph node
